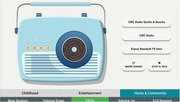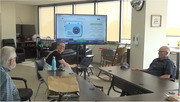# Feasibility Testing the Computer Interactive Reminiscing and Conversation Aid in Canada

**DOI:** 10.1002/alz70863_110564

**Published:** 2025-12-23

**Authors:** Arlene Astell, Erica Dove, Shoshana Green, Rushil Joshi, Rachel Zuo, Megan E O'Connell

**Affiliations:** ^1^ University of Toronto, Toronto, ON Canada; ^2^ KITE Research Institute, Toronto Rehabilitation Institute, University Health Network, Toronto, ON Canada; ^3^ University of Saskatchewan, Saskatoon, SK Canada

## Abstract

**Background:**

The Computer Interactive Reminiscing and Conversation Aid (CIRCA) is a multimedia conversation support for people living with dementia and caregivers, comprising generic photographs, music, and video clips (e.g., famous songs)^1^. CIRCA provides a meaningful shared activity for people with dementia and caregivers, which can positively impact cognition and quality of life^2^. Originally developed in the UK as an offline touchscreen resource, the need for a refreshed version aligned with current digital services and resources was identified. This feasibility study tested a new version of CIRCA containing a broad selection of Canadian‐relevant contents.

**Method:**

People with dementia, care staff, and family caregivers were recruited from community‐based organizations in Ontario and Saskatchewan, Canada. Staff received training to deliver CIRCA as a group program, which their clients could also use at home with their families. During the group sessions, video recordings captured participants’ responses to the contents. Usage data (e.g., date, time, duration) was captured on the back‐end. At the end of the study, interviews and focus groups captured satisfaction, feedback, suggestions for additional contents, and perceptions of CIRCA's feasibility for Canadians with dementia.

**Result:**

To date, 49 CIRCA sessions have been run with 19 staff (19F/0M), 4 family caregivers (3F/1M), and 69 clients (38F/31M) across six locations and in four family homes. Session length ranged from 6‐82 minutes, with a mean duration of 32.25 minutes. Canadian CIRCA contents (e.g., videos of Ontario Place) have sparked a variety of conversations, laughs, and memories, with both clients and facilitators enjoying the experience.

**Conclusion:**

People with dementia and their caregivers enjoyed using CIRCA, including shared exploration of CIRCA contents, increased time spent in conversation, sharing of stories from the past, and positive interactions. The results support potential scaling of CIRCA for Canadians living with dementia